# An Improved Surgical Technique to Increase Neck Width of Elastase-Induced Aneurysm Model in Rabbits: A Prospective Study

**DOI:** 10.3389/fneur.2022.889140

**Published:** 2022-07-01

**Authors:** Yang Zhang, Yu He, ChaoJie Tang, YuFan Wu, Yi Gu, BinXian Gu, Li Chen, WenWei Gao, ZhiGuo Zhou, YouKe Qi, FaJiang Mao, YongNing Sun, Wu Wang

**Affiliations:** ^1^Institute of Diagnostic and Interventional Radiology, Shanghai Jiao Tong University Affiliated Sixth People's Hospital, Shanghai, China; ^2^Neurosurgery, Shanghai Jiao Tong University Affiliated Sixth People's Hospital, Shanghai, China; ^3^Joint International Research Laboratory of Resource Chemistry, Ministry of Education, Shanghai Normal University, Shanghai, China; ^4^Department of Cardiology, Shanghai Municipal Hospital of Tradition Chinese Medicine, Shanghai University of Traditional Chinese Medicine, Shanghai, China

**Keywords:** aneurysm neck width, elastase, temporary aneurysm clips, rabbit, aneurysm model

## Abstract

**Background:**

Rabbit elastase-induced aneurysms have widely been used to test various endovascular materials over the past two decades. However, wide-necked aneurysms cannot be stably constructed.

**Objective:**

The purpose of the study was to increase the neck width of the elastase-induced aneurysm model in rabbits *via* an improved surgical technique with two temporary clips.

**Materials and Methods:**

Fifty-four elastase-induced aneurysms in rabbits were successfully created. Group 1 was (*n* = 34) composed of cases in which two temporary aneurysm clips were placed closely medially and laterally to the origin of the right common carotid artery (RCCA), respectively. Group 2 (*n* = 20) included cases in which a single temporary aneurysm clip was placed crossed the origin of RCCA. Digital subtraction angiography (DSA) was performed before and immediately after elastase incubation and 3 weeks later. The diameter of the origin of RCCA before and immediately after elastase incubation and aneurysm sizes of the two groups were measured and compared. Moreover, the correlation analysis was performed between the diameter of the origin of RCCA immediately after elastase incubation and aneurysm neck width.

**Results:**

The mean aneurysm neck and dome width of group 1 were both significantly larger than that of group 2 (*p*-value < 0.001 and *p*-value = 0.005, respectively). Moreover, the proportion of wide-necked aneurysms (neck width ≥4 mm) in group 1 was significantly larger than that in group 2 (*p*-value = 0.004) and the mean dome to neck ratio (D/N) of group 1 was smaller than that of group 2 (*p*-value = 0.008). Furthermore, there was a positive correlation between the diameter of the origin of RCCA immediately after elastase incubation and aneurysm neck width.

**Conclusion:**

The improved surgical technique with two temporary clips, focusing on the direct contact of elastase with the origin of RCCA, could increase the neck width of elastase-induced aneurysm models in rabbits.

## Introduction

The preclinical experiment studies play an important role in helping practitioners and device developers improve techniques and tools for endovascular treatment (EVT) of intracranial aneurysms. Elastase-induced aneurysm of the right common carotid artery (RCCA) in rabbits has been widely used to test the safety and efficacy of new endovascular devices prior to use in humans (1–6). The previous preclinical studies have indicated that the elastase-induced aneurysm model has similar hemodynamic, morphological, and histologic characteristics to human unruptured aneurysms, and demonstrated similar healing responses and biologic characteristics to treat with coils, stents, and flow diverters as human aneurysms (7–10). The aneurysm model is created through a combination of surgical and endovascular techniques, which have been first described by Kallmes and Altes et al. from Mayo clinic (1, 11). Since then, many investigators have been practicing reliable modifications by altering the volume and configuration, including the neck width, of the aneurysm model to test new endovascular devices (10, 12–18). To our knowledge, the origin of RCCA (the neck of the aneurysm model) digested sufficiently by elastase is a critical factor to improve the neck width of the elastase-induced aneurysm model.

**Figure 1:**
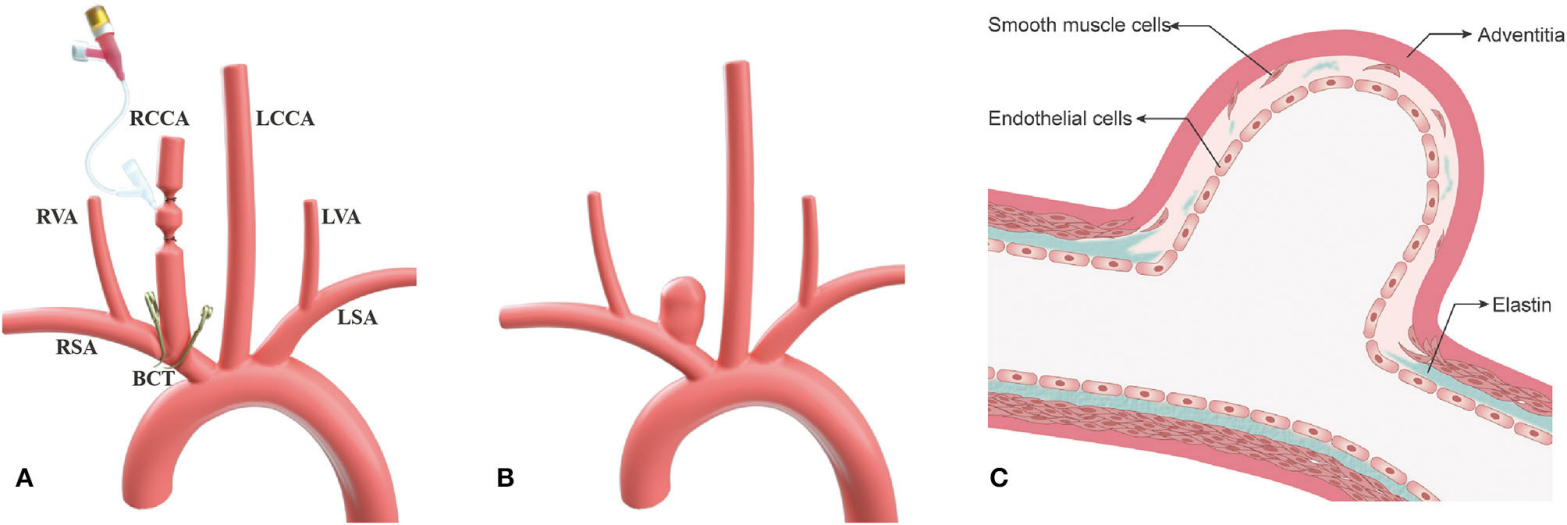
The improved surgical technique, two temporary aneurysm clips technique, for aneurysm model in rabbits. **(A)** Schematic picture depicting an improved technique for increasing neck width of the aneurysm at the right common carotid artery (RCCA); **(B)** Illustration depicting wide-necked aneurysm at the RCCA after 3-week follow-up; and **(C)** Schematic illustration displaying sufficient elastin digestion at the aneurysm neck and dome. RCCA, right common carotid artery; LCCA, left common carotid artery; RSA, right subclavian artery; LSA, light subclavian artery; RVA, right vertebral artery; LVA, left vertebral artery; BCT, brachiocephalic trunk.

According to preliminary experiment practice (10, 14), we found that when a single temporary arcuated aneurysm clip was used across the origin of RCCA to create an aneurysm model, the neck width was not as large as we expect. Thus, we tended to modify the procedure by placing two temporary aneurysm clips closely medially and laterally to the origin of RCCA, respectively to achieve complete digestion of the elastin at the origin of RCCA (Figure 1) and compare the aneurysm parameters between the improved technique and the traditional single temporary aneurysm clip technique to check whether the improved technique could increase aneurysm neck width.

## Materials and Methods

### Aneurysm Creation

**Figure 2:**
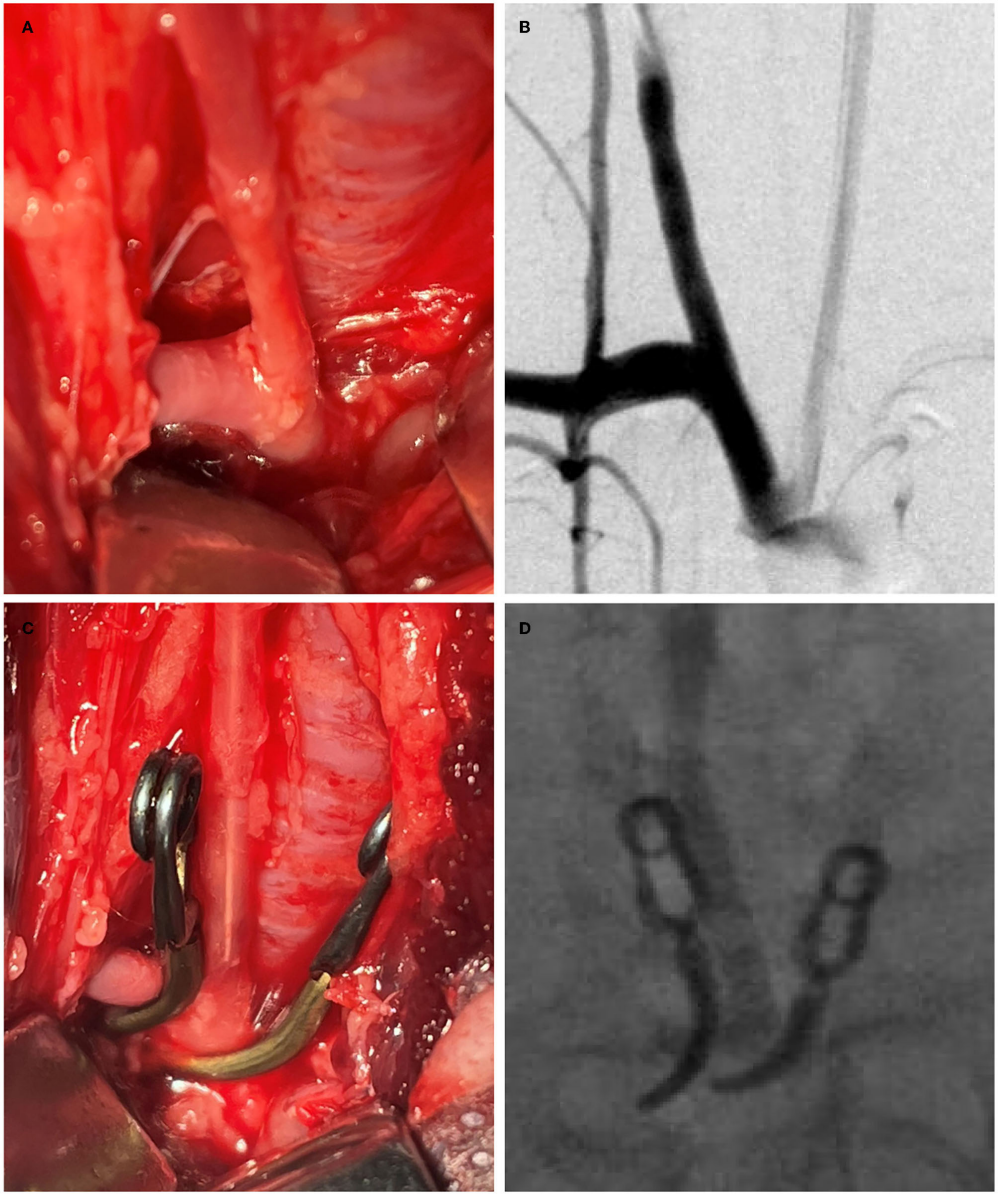
The process of the improved surgical technique with two temporary clips. **(A)** The origin of the right common carotid artery (RCCA) at its junction with the subclavian and brachiocephalic arteries was exposed; **(B)** Angiography shows the subclavian artery, the brachiocephalic artery, and the RCCA *via* a 20-gauge intravenous catheter; **(C)** Two temporary aneurysm clips were placed closely medially and laterally, respectively to the origin of RCCA which was inserted by a 20-gauge intravenous catheter; and **(D)** Under fluoroscopy, the origin of RCCA was confirmed that it can be filled with elastase mixed with a contrast agent without leakage.

All experimental procedures were performed in accordance with the National Institutes of Health guidelines for humane handling of animals and were approved by the animal research committee of Shanghai Jiao Tong University Affiliated Sixth People's Hospital. All rabbits (body weight, 2.50~3.00 kg) were maintained on a standard laboratory diet. In the study, the details of improved surgical technique are as follows (group 1) (Figures 1A, 2). After sufficient anesthesia was induced with a single administration of intramuscular injection of 1% sodium pentobarbital in 1 mg/kg, hairs around the incision were shaved, which was located at the right of the midline from the thyroid cartilage to the manubrium sterni. The animals breathed spontaneously during the surgery. The detailed procedures had been described in the previous literature (10, 14). Following a 6–8 cm incision was made, muscles were separated and removed layer by layer. The trunk and the origin of RCCA at its junction with the subclavian and the brachiocephalic arteries were exposed and isolated (Figure 2A). After the RCCA was ligated distally, a 20-gauge intravenous catheter was advanced retrograde into the RCCA, and a 3–0 silk ligature was used to ligate the proximal to the cannulation site. Digital subtraction angiography (DSA, AXIOM Artis dBA; Siemens Medical Solutions, Germany) via the catheter showed the arteries, including the right subclavian artery, the brachiocephalic trunk, and the RCCA (Figure 2B). The diameter of the origin of RCCA on the DSA images before elastase incubation was measured in reference to the external sizing device. Then, two temporary aneurysm clips were successively placed closely medially and laterally to the origin of RCCA, respectively (Figures 1A, 2C), and DSA *via* the catheter confirmed the complete occlusion of the right brachiocephalic artery and the right subclavian artery. More importantly, the origin of RCCA and the junction of the RCCA with the subclavian and brachiocephalic trunk could be filled with a contrast medium under fluoroscopy for at least 2 min to confirm that the elastase could directly contact with the origin of RCCA without leakage. Subsequently, remaining blood within the isolated RCCA segment was aspirated, and 80 units of porcine pancreatic elastase (Worthington Biochemical Corp., Lakewood, NJ) mixed with equivalent amounts of nonionic contrast-material were infused into the lumen of the isolated RCCA segment and allowed to incubate for 20 min (Figure 2D). After incubation, the elastase solution and two temporary aneurysm clips were removed, successively. We also measured the diameter of the origin of RCCA on the DSA images immediately after elastase incubation. Then a 3–0 silk ligature was tied about 15 mm distally from the origin of RCCA (18). At last, the RCCA was transected just distal to the last ligation, and the wound was closed in multiple layers with 3–0 Vicryl. After the operation, all rabbits were fed with adequate water and food in cages. The rabbits in group 2 underwent the same procedure as those in group 1, except that two temporary aneurysm clips were replaced by a single temporary aneurysm clip which was placed across the origin of RCCA (10, 14).

### Follow-Up Angiography

DSA was performed to confirm the aneurysm models 3 weeks after creation. The animals were anesthetized as described above. Using a sterile technique, surgical exposure of the right common femoral artery was performed, and a 5 F vascular sheath was placed. Heparin (100 U/kg) was administered intravenously. A 5 F catheter (Envoy; Cordis Endovascular) was advanced into the aortic arch to perform DSA. The neck width, dome height, and dome width of the aneurysm models were measured. A wide-necked aneurysm was characterized as neck width ≥4 mm (19).

### Statistical Analysis

Two observers (YG and BG with 10 and 20 neurointerventional experience, respectively) who were not clear about the grouping detail measured all data independently. Interobserver variability was measured by using intraclass correlation (ICC). ICC of <0.4 indicates poor reproducibility, ICC between 0.4 and 0.75 indicates fair to good reproducibility, and ≥0.75 indicates excellent reproducibility. Continuous data are presented as mean ± SD. Student's *t*-test was used for normally distributed data, and the Mann–Whitney U test was used for data that were not normally distributed. Categorical data were expressed as frequencies and group percentages and tested by Chi-squared and Fisher's exact tests. *p*-values<0.05 were considered statistically significant. A regression analysis was performed for the relationship between the diameter of the origin of RCCA immediately after elastase incubation and the aneurysm neck width. Statistical analysis was performed using SPSS 26.0 (SPSS Inc, Chicago, IL, USA).

### Histology

The animals were sacrificed to obtain the specimens, including the right brachiocephalic trunk, RCCA aneurysm and part of the ascending aorta, the descending aorta, the right subclavian artery, and the left CCA. The specimen was flushed with saline and fixed in 10% formaldehyde for more than 24 h. Specimens were embedded in paraffin, sectioned, and stained with hematoxylin-eosin (H&E) and Verhoeff's Van Gieson stain.

## Results

The elastase-induced aneurysm models in group 1 and group 2 were successfully created in 34 of 37 and 20 of 22 rabbits, respectively. Three and two rabbits in group 1 and group 2 died, respectively due to iatrogenic pneumothorax and bleeding during the experiment procedure.

**Figure 3:**
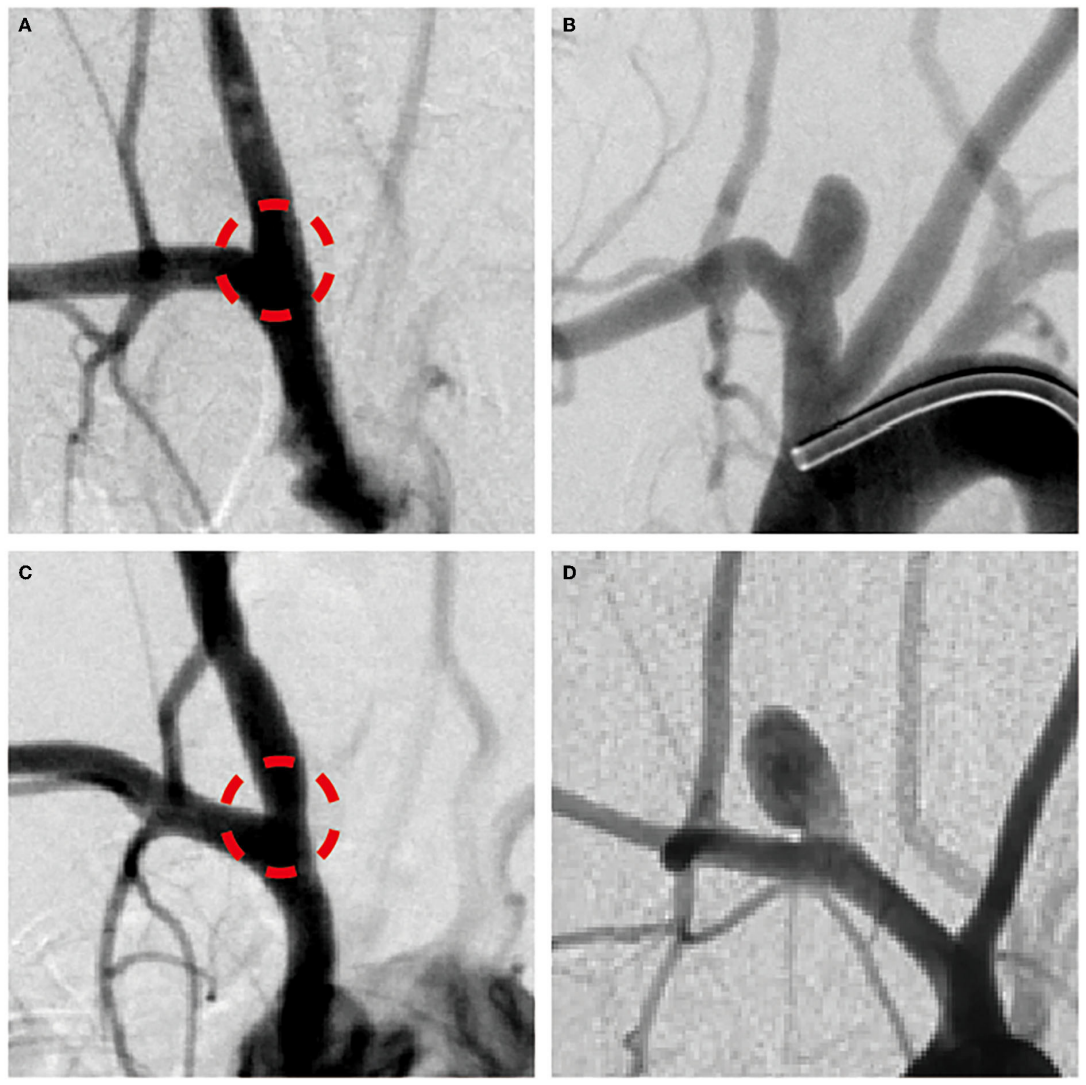
**(A,C)** The angiogram immediately after right common carotid artery (RCCA) incubated by elastase and **(B,D)** 3-week follow-up angiogram to confirm the creation of RCCA aneurysm model in rabbits of groups 1 and 2, respectively.

**Figure 4:**
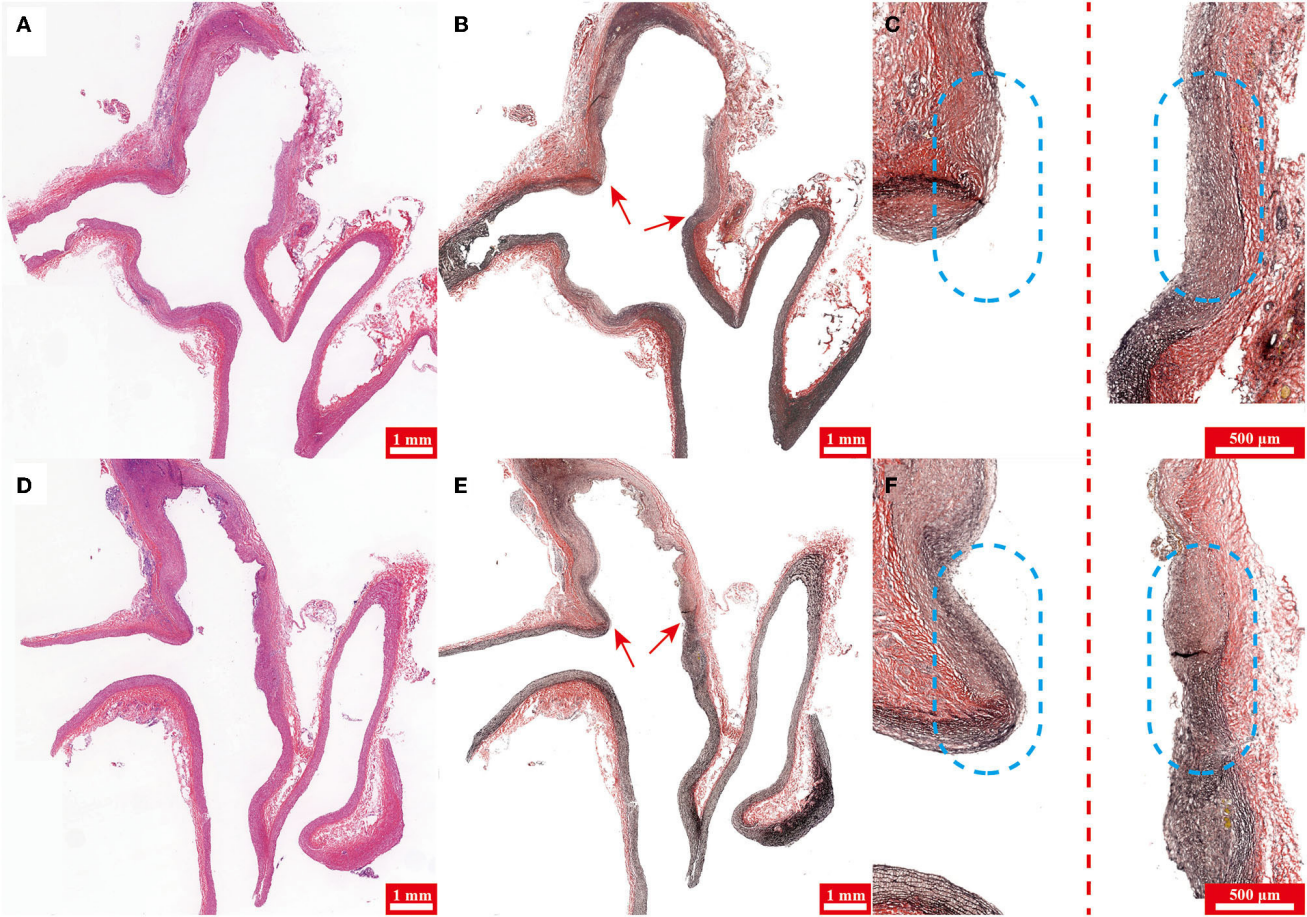
**(A,D)** Hematoxylin-eosin (H&E) staining and **(B,E)** Verhoeff's Van Gieson stain of specimens harvested from rabbits in groups 1 and 2, respectively. **(C,F)** Magnified and pieced pictures of **(B,E)** respectively. Note the aneurysm neck (arrow) in **(B,E)**. The left and right sides of the dotted line are the medial and lateral parts of specimens, respectively in **(C,F)** and the dashed circles highlight the aneurysm neck.

Three weeks later, DSA and histological analysis were performed in all rabbits without mortality. The diameter of the origin of RCCA before and immediately after elastase incubation, aneurysm sizes, and the proportion of wide-necked aneurysm are presented in Tables 1, 2. Representative images are shown in Figures 3, 4.

**Table 1:** The diameter of the origin of the right common carotid artery (RCCA) before and immediately after elastase incubation and aneurysm sizes in groups 1 and 2.

**Variables**	**Group 1**	**Group 2**	***P*-value**
The diameter of the origin of RCCA before elastase incubation, mm (mean ± SD)	2.06 ± 0.18	2.05 ± 0.17	0.88
The diameter of the origin of RCCA immediately after elastase incubation, mm (mean ± SD)	2.87 ± 0.49	2.47 ± 0.58	0.078
Dome height, mm (mean ± SD)	6.38 ± 1.82	5.52 ± 1.39	0.07
Dome width, mm (mean ± SD)	4.25 ± 1.12	3.44 ± 0.59	0.005
Neck width, mm (mean ± SD)	4.09 ± 0.80	2.85 ± 0.69	<0.001
Dome to neck ratio (mean ± SD)	1.59 ± 0.45	1.99 ± 0.55	0.008

**Table 2:** Frequency of aneurysm neck width ≥4 mm in groups 1 and 2.

**Neck width**	**Group 1**	**Group 2**	***P*-value**
≥4 mm, *n* (%)	14 (41.2%)	1 (5.0%)	0.004
<4 mm, *n* (%)	20 (58.8%)	19 (95.0%)	

### Interobserver Reproducibility

The interobserver reproducibility of all measurements was excellent. The diameter of the origin of RCCA before and immediately after elastase incubation ICC was 0.987 and 0.988, respectively; aneurysm dome height ICC, 0.981; aneurysm dome width ICC, 0.984; aneurysm neck width ICC, 0.990.

### The Diameter of the Origin of RCCA Before Elastase Incubation

The mean diameters of the origin of RCCA before elastase incubation for group 1 and 2 were 2.06 ± 0.18 (range, 1.79 to 2.38 mm), and 2.05 ± 0.17 (range, 1.84 to 2.29 mm), respectively (*p*-value = 0.88).

### The Diameter of the Origin of RCCA Immediately After Elastase Incubation

The mean diameters of the origin of RCCA immediately after elastase incubation for group 1 and 2 were 2.87 ± 0.49 (range, 2.02 to 3.90 mm), and 2.47 ± 0.58 (range 1.97 to 3.45 mm), respectively (*p*-value = 0.078).

### Aneurysm Dome Height

The mean dome heights for group 1 and 2 were 6.38 ± 1.82 (range, 3.23 to 12.13 mm), and 5.52 ± 1.39 (range, 2.90 to 8.19 mm), respectively (*p-*value = 0.07).

### Aneurysm Dome Width

The mean dome widths for group 1 and 2 were 4.25 ± 1.12 (range, 2.62 to 7.36 mm) and 3.44 ± 0.59 (range, 2.63 to 4.72 mm), respectively (*p*-value = 0.005).

### Aneurysm Neck Width

The mean neck widths for group 1 and 2 were 4.09 ± 0.80 (range, 2.78 to 6.36 mm) and 2.85 ± 0.69 (range, 1.97 to 4.55 mm), respectively (*p*-value < 0.001). Fourteen (41.2%) of 34 aneurysms in group 1 could be attributed to wide-necked and there were only 1 wide-necked aneurysm (5.0%) in group 2 (*n* = 20) (*p*-value = 0.004).

### Dome to Neck Ratio

The mean dome to neck ratio (D/N) for group 1 and 2 were 1.59 ± 0.45 (range, 1.06 to 3.02) and 1.99 ± 0.55 (range, 1.06 to 3.08), respectively (*p*-value = 0.007).

### Histology

Hematoxylin-eosin staining of specimens harvested from rabbits in groups 1 and 2 demonstrated the aneurysm were both successfully created in two groups (Figures 4A,D). Verhoeff's Van Gieson stain (stains for elastin) of specimens showed that the aneurysm dome wall lacked elastin in both groups 1 and 2 (Figures 4B,E), however, compared with group 2, elastin at the aneurysm neck in group 1 was digested more sufficiently (Figures 4C,F).

### Correlation Analysis

**Figure 5:**
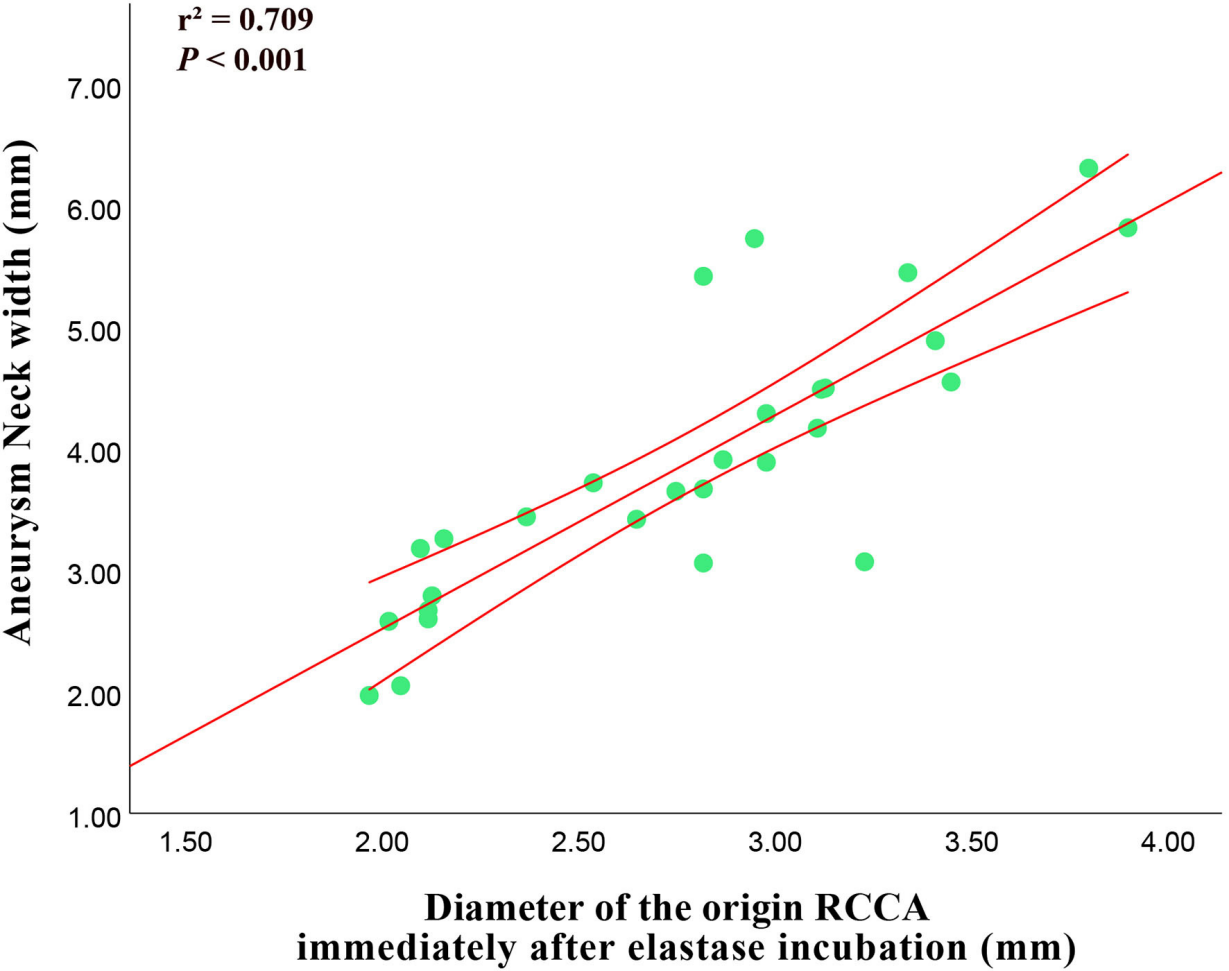
Correlation between the diameter of the origin of RCCA immediately after elastase incubation and 3-week follow-up aneurysm neck width.

The diameter of the origin of RCCA immediately after elastase incubation had positive correlation with aneurysm neck width (*r*^2^ = 0.709, *p*-value < 0.001) (Figure 5).

## Discussion

The New Zealand rabbit elastase-induced aneurysm of the RCCA is one of the most common experiment models in testing the safety and efficacy of new endovascular devices in the field of interventional neuroradiology and biomaterial engineering (1–6). However, the configurations of the aneurysm, especially neck width, still remain randomized and uncontrollable. In the study, the aneurysm clip technology was modified, and compared with the improved technology, two temporary aneurysm clips, with the traditional single clip technique to prove whether the improved technique can reproducibly increase aneurysm neck width. When two temporary aneurysm clips were placed closely medially and laterally to the origin of RCCA, respectively, the elastin at the origin of RCCA could be completely digested and the aneurysm neck size could be relatively wide.

Because the weight of rabbits was strictly controlled, the mean diameters of the origin of RCCA before elastase incubation for two groups were almost the same, which could avoid the original RCCA diameter effect on the aneurysm neck width. Besides, though not statistically significant, after elastase digestion, the diameter of the origin of RCCA of group 1 was larger than that of group 2 (*p*-value = 0.078). The mean aneurysm dome height and width of group 1 were larger than those of group 2, moreover, the difference in dome width had statistical significance (*p*-value = 0.005) and that of dome height nearly reached statistical significance (*p*-value = 0.07). This phenomenon seemed to be similar to the aneurysm in humans. The aneurysm neck width had a positive correlation between the dome width and the neck height (20). Meanwhile, the D/N, another index used to evaluate wide-necked aneurysms in a clinic, of group 1 was significantly smaller than that of group 2 (*p*-value = 0.008).

In addition, a strong positive correlation was observed between the diameters of the origin of RCCA immediately after elastase incubation and the aneurysm neck width, which was similar to that reported by Onizuka et al. (21). Compared with preliminary technology, vein-pouch technology, the histology of aneurysms created by elastase bore a closer resemblance to that of human aneurysms, however, the aneurysm needed about 3 weeks to mature. Based on the correlation between the diameters of the origin of RCCA immediately after elastase incubation and the aneurysm neck width, researchers could predict the final aneurysm morphology, thus, may save time and reduce cost.

Since EVT was used to treat intracranial aneurysms, complete aneurysm occlusion is often difficult to achieve in wide-necked aneurysms. For testing, the developed techniques and endovascular devices require animal models that closely mimic technically hazardous aneurysm configurations, especially wide-necked aneurysms. Ding et al. reported the method to improve neck size *via* the lower position of the balloon to achieve the origin of RCCA digested sufficiently by elastase (16, 17). However, inflation of the proximal balloon at the origin of the RCCA may denude the internal vessel lining of the brachiocephalic artery and the origin of the RCCA introducing histologic changes (10). So aneurysm clip technique to preserve histology was preferred. Moreover, in the study, we showed that two temporary aneurysm clips could also achieve sufficient digestion of elastin at the origin of RCCA, which is similar to the method of Ding et al. Besides, the mean neck width and proportion of wide-neck aneurysm of improved technique are also close to those of their method (4.09 ± 0.80 vs. 3.93 ± 0.91; 41.2 vs. 46%, respectively) (17).

In addition, the improved technique is superior to the traditional single temporary aneurysm clip technique in increasing the neck width of aneurysms, which can be attributed to the following reasons: (1) A single clip is easy to cover the origin of the carotid artery because the width of aneurysm clip blade was approximately 1 mm. In order to fully digest the elastin at the origin of RCCA, the clip needs to be placed more accurately, which is also closely related to the relationship between the arterial anatomy and the shape of the clip. On the contrary, the improved technique is less affected by experience and anatomy. (2) The single clip technique needs to place the clip horizontally, which takes up a lot of space. Due to the extrusion of the tissue, it is easy to cause the displacement of the clip after location, however, in improved technique, the clip is placed longitudinally to avoid displacement.

One shortcoming of the improved technique is that elastase might digest elastin of the parent artery causing dilation. It mainly occurred in the first third of group 1 (6 of 13, 46.2%). With the improvement of experience, the head of the aneurysm clips was put closer to reduce the damage to the parent artery, therefore, the probability of parent artery elastin injury in the middle third and the last third gradually decreased (3 of 12, 25.0% and 1 of 12, 8.3%, respectively). Meanwhile, group 2 had less parent artery dilation (2 of 20, 10%). This shortcoming was common in the previous studies which were also in order to increase the neck width of aneurysms (16, 17). However, it was proved in the present study that with the improvement of experience, this defect could be avoided.

Moreover, the technique of surgically exposing the origin of the RCCA could precisely place two temporary aneurysm clips and another advantage of our technique is that it is easy to distinguish and isolate aberrant origins of the superior thyroid artery and the tracheoesophageal branch from the RCCA to avoid elastase leakage and reduce mortality (22). Additionally, fluoroscopy could confirm that elastin at the origin of RCCA is directly digested without leakage. Furthermore, the aneurysm creation procedure is quite simple and easy to perform for training for the interventional procedures. Novices can adapt our technology in a short period of time and achieve highly reproducible aneurysms with large neck width for evaluating endovascular devices and techniques for aneurysmal therapy.

## Innovations and Limitations

Although the prospective study shows that improving the surgical temporary clips technique can increase neck width of morphologically reproducible elastase-induced aneurysm model in rabbits, there have been some limitations that should be acknowledged. The study includes small subjects and angiography was followed up for only 3-weeks, so additional long-term studies with large sample sizes are needed to determine whether the improved technique can increase the neck width of the aneurysm model more reliably than a single clip technique. Moreover, though angiographic projections parallel to the aneurysm plane have been obtained to the best of our ability, some small projection variations are inevitable. Last but not least, exact aneurysm size and neck width are also subject to interpretation errors. In the study, two observers were used to diminish this error.

## Conclusion

The improved surgical temporary clips technique, focusing on the direct contact of elastase with the origin of RCCA, to create a morphologically reproducible elastase-induced relatively wide neck aneurysm model in rabbits is feasible. Large cohort studies and long-term follow-up are needed to further clarify the efficacy of the technique.

## Data Availability Statement

The original contributions presented in the study are included in the article/supplementary material, further inquiries can be directed to the corresponding author/s.

## Ethics Statement

The animal study was reviewed and approved by the Animal Research Committee of Shanghai Jiao Tong University Affiliated Sixth People's Hospital.

## Author Contributions

YZ, YH, and WW participated in designing the study, collecting data, analyzing the data, and writing the article. CT, YW, LC, YG, and BG were responsible for data collection and processing. YZ and FM did the statistical analysis. YS, YQ, and ZZ made critical revisions to the manuscript for important intellectual content. YS and WW were responsible for study supervision and organization of the project. WW accepts full responsibility for the finished work. All authors contributed to the article and approved the submitted version.

## Funding

This study was partially supported by the National Natural Science Foundation of China (81771951); Shanghai Pujiang Program (2020PJD043); Shanghai Science and Technology Innovation Project (201409006000); and the Foundation of MicroPort Medical Engineering (MP2021Q1C016).

## Conflict of Interest

The authors declare that the research was conducted in the absence of any commercial or financial relationships that could be construed as a potential conflict of interest.

## Publisher's Note

All claims expressed in this article are solely those of the authors and do not necessarily represent those of their affiliated organizations, or those of the publisher, the editors and the reviewers. Any product that may be evaluated in this article, or claim that may be made by its manufacturer, is not guaranteed or endorsed by the publisher.

## References

[1] Kallmes DF Helm GA Hudson SB Altes TA Do HM Mandell JW. histologic evaluation of platinum coil embolization in an aneurysm model in rabbits. Radiology. (1999) 213:217–22. 10.1148/radiology.213.1.r99oc16217 10540665

[2] Krings T Busch C Sellhaus B Drexler AY Bovi M Hermanns-Sachweh B. Long-term histological and scanning electron microscopy results of endovascular and operative treatments of experimentally induced aneurysms in the rabbit. Neurosurgery. (2006) 59:911–24. 10.1227/01.NEU.0000232841.08876.DA 17038956

[3] Kallmes DF Ding YH Dai D Kadirvel R Lewis DA Cloft HJ. A second-generation, endoluminal, flow-disrupting device for treatment of saccular aneurysms. AJNR Am J Neuroradiol. (2009) 30:1153–8. 10.3174/ajnr.A1530 19369609 PMC7051356

[4] Ding Y Dai D Kallmes DF Schroeder D Kealey CP Gupta V. Preclinical testing of a novel thin film nitinol flow-diversion stent in a rabbit elastase aneurysm model. AJNR Am J Neuroradiol. (2016) 37:497–501. 10.3174/ajnr.A4568 26494695 PMC4792643

[5] Marosfoi M Langan ET Strittmatter L van der Marel K Vedantham S AJ Lylyk IR. In situ tissue engineering: endothelial growth patterns as a function of flow diverter design. J Neurointerv Surg. (2017) 9:994–8. 10.1136/neurintsurg-2016-012669 27707872

[6] Nishi H Ishii A Ono I Abekura Y Ikeda H Arai D. Biodegradable flow diverter for the treatment of intracranial aneurysms: a pilot study using a rabbit aneurysm model. J Am Heart Assoc. (2019) 8:e014074. 10.1161/JAHA.119.014074 31583935 PMC6818033

[7] Short JG Fujiwara NH Marx WF Helm GA Cloft HJ Kallmes DF. Elastase-induced saccular aneurysms in rabbits: comparison of geometric features with those of human aneurysms. AJNR Am J Neuroradiol. (2001) 22:1833–7. 11733310 PMC7973827

[8] Zeng Z Kallmes DF Durka MJ Ding Y Lewis D KR Robertson AM. Hemodynamics and anatomy of elastase-induced rabbit aneurysm models: similarity to human cerebral aneurysms? AJNR Am J Neuroradiol. (2011) 32:595–601. 10.3174/ajnr.A2324 21273353 PMC3920548

[9] Wang S Dai D Kolumam Parameswaran P Kadirvel R Ding YH Robertson AM. Rabbit aneurysm models mimic histologic wall types identified in human intracranial aneurysms. J Neurointerv Surg. (2018) 10:411–5. 10.1136/neurintsurg-2017-013264 28768819 PMC5796872

[10] Hoh BL Rabinov JD Pryor JC Ogilvy CS A Modified technique for using elastase to create saccular aneurysms in animals that histologically and hemodynamically resemble aneurysms in human. Acta Neurochir. (2004) 146:705–11. 10.1007/s00701-004-0276-6 15197614

[11] Altes TA Cloft HJ Short JG DeGast A Do HM Helm GA. 1999 ARRS Executive Council Award. Creation of saccular aneurysms in the rabbit: a model suitable for testing endovascular devices American Roentgen Ray Society. AJR Am J Roentgenol. (2000) 174:349–54. 10.2214/ajr.174.2.1740349 10658703

[12] Krings T Möller-Hartmann W Hans FJ Thiex R Brunn A Scherer K. A refined method for creating saccular aneurysms in the rabbit. Neuroradiology. (2003) 45:423–9. 10.1007/s00234-003-0976-2 12774180

[13] Ding YH Danielson MA Kadirvel R Dai D Lewis DA Cloft HJ. Modified technique to create morphologically reproducible elastase-induced aneurysms in rabbits. Neuroradiology. (2006) 48:528–32. 10.1007/s00234-006-0093-0 16708202

[14] Wang K Huang Q Hong B Xu Y Zhao W Chen J. Neck Injury Is Critical to Elastase-Induced Aneurysm Model. AJNR Am J Neuroradiol. (2009) 30:1685–7. 10.3174/ajnr.A1542 19386733 PMC7051499

[15] Thiex R Möller-Hartmann W Hans FJ Scherer K Krings T. Are the configuration and neck morphology of experimental aneurysms predictable? A technical approach. Neuroradiology. (2004) 46:571–6. 10.1007/s00234-004-1218-y 15258710

[16] Ding YH Dai D Lewis DA Danielson MA Kadirvel R Mandrekar JN. Can neck size in elastase-induced aneurysms be controlled? A prospective study. AJNR Am J Neuroradiol. (2005) 26:2364–7. 16219846 PMC7976130

[17] Ding YH Dai D Lewis DA Danielson MA Kadirvel R Mandrekar JN. Can neck size in elastase-induced aneurysms be controlled? A retrospective study. AJNR Am J Neuroradiol. (2006) 27:1681–4. 16971614 PMC8139774

[18] Ding YH Dai D Danielson MA Kadirvel R Lewis DA Cloft HJ. Control of aneurysm volume by adjusting the position of ligation during creation of elastase-induced aneurysms: a prospective study. AJNR Am J Neuroradiol. (2007) 28:857–9. 17494656 PMC8134338

[19] Brinjikji W Cloft HJ Kallmes DF. Difficult aneurysms for endovascular treatment: overwide or undertall? AJNR Am J Neuroradiol. (2009) 30:1513–7. 10.3174/ajnr.A1633 19461057 PMC7051599

[20] Parlea L Fahrig R Holdsworth DW Lownie SP. An analysis of the geometry of saccular intracranial aneurysms. AJNR Am J Neuroradiol. (1999) 20:1079–89. 10445447 PMC7056259

[21] Onizuka M Miskolczi L Gounis MJ Seong J Lieber BB Wakhloo AK. Elastase-induced aneurysms in rabbits: effect of postconstruction geometry on final size. AJNR Am J Neuroradiol. (2006) 27:1129–31. 16687557 PMC7975736

[22] Lewis DA Ding YH Dai D Kadirvel R Danielson MA Cloft HJ. Morbidity and mortality associated with creation of elastase-induced saccular aneurysms in a rabbit model. AJNR Am J Neuroradiol. (2009) 30:91–4. 10.3174/ajnr.A1369 19001536 PMC2626645

